# An Experimental and Theoretical Study of Impact of Device Parameters on Performance of AlN/Sapphire-Based SAW Temperature Sensors

**DOI:** 10.3390/mi13010040

**Published:** 2021-12-28

**Authors:** Hongrui Lv, Yinglong Huang, Yujie Ai, Zhe Liu, Defeng Lin, Zhe Cheng, Lifang Jia, Bingliang Guo, Boyu Dong, Yun Zhang

**Affiliations:** 1 Laboratory of Solid State Optoelectronics Information Technology, Institute of Semiconductors, CAS, Beijing 100083, China; lvhongrui@semi.ac.cn (H.L.); liuzhe@semi.ac.cn (Z.L.); dflin@semi.ac.cn (D.L.); zhecheng@semi.ac.cn (Z.C.); lfjia@semi.ac.cn (L.J.); 2 Center of Materials Science and Optoelectronics Engineering, University of Chinese Academy of Sciences, Beijing 100049, China; 3 China Electronics Standardization Institute, Beijing 100007, China; huangyl@cesi.cn; 4 Lishui Zhongke Semiconductor Material Co., Ltd., Lishui 323000, China; 5 NAURA Technology Group Co., Ltd., Beijing 100176, China; guobingliang@naura.com (B.G.); boyu.dong@naura.com (B.D.)

**Keywords:** AlN film, surface acoustic wave, temperature sensor, acoustic propagation direction, temperature coefficient of frequency, quality factor

## Abstract

The impact of device parameters, including AlN film thickness (*h*_AlN_), number of interdigital transducers (*N*_IDT_), and acoustic propagation direction, on the performance of *c*-plane AlN/sapphire-based SAW temperature sensors with an acoustic wavelength (*λ*) of 8 μm, was investigated. The results showed that resonant frequency (*f*_r_) decreased linearly, the quality factor (*Q*) decreased and the electromechanical coupling coefficient $\left(K_{t}^{2}\right)$ increased for all the sensors with temperature increasing from −50 to 250 °C. The temperature coefficients of frequency (TCFs) of sensors on AlN films with thicknesses of 0.8 and 1.2 μm were −65.57 and −62.49 ppm/°C, respectively, indicating that a reduction in *h*_AlN_/*λ* favored the improvement of TCF. The acoustic propagation direction and *N*_IDT_ did not obviously impact the TCF of sensors, but they significantly influenced the *Q* and $K_{t}^{2}$ of the sensors. At all temperatures measured, sensors along the *a*-direction exhibited higher *f*_r_, *Q* and $K_{t}^{2}$ than those along the *m*-direction, and sensors with *N*_IDT_ of 300 showed higher *Q* and $K_{t}^{2}$ values than those with *N*_IDT_ of 100 and 180. Moreover, the elastic stiffness of AlN was extracted by fitting coupling of modes (COM) model simulation to the experimental results of sensors along different directions considering Euler transformation of material parameter-tensors. The higher *f*_r_ of the sensor along the *a*-direction than that along the *m*-direction can be attributed to its larger elastic stiffness *c*_11_, *c*_22_, *c*_44_, and *c*_55_ values.

## 1. Introduction

AlN-based surface acoustic wave (SAW) sensors have attracted considerable attention for wireless temperature sensing, as they are well suited for harsh environments or for placement on rotating machinery [1,2,3]. Compared with traditional piezoelectric materials, including LiNbO_3_, LiTaO_3_ and quartz with maximum working temperature much lower than 600 °C, AlN is very promising for the fabrication of high temperature sensors because it remains stable at 1000 °C [4]. There have been reports of AlN-based SAW temperature sensors on Si [5,6], sapphire [7,8], and silicon carbide [9,10], but AlN/sapphire-based sensors have attracted the most attention because of their good thermal stability, high acoustic velocity, high quality factor (*Q*) and low cost. Until now, most studies have focused on improving the working temperature of AlN-based SAW sensors [11,12,13]; however, the basic performance of these sensors still requires further improvement for their commercialization. As a result, a systematic study on the impact of device structure on the performance of AlN/sapphire-based SAW temperature sensors, which has rarely been reported, is necessary to further improve the performance of these devices.

In this paper, we investigated the impact of device parameters including AlN film thickness (*h*_AlN_), number of interdigital transducers (*N*_IDT_), and acoustic propagation direction on the temperature coefficient of frequency (TCF), the quality factor (*Q*), and the effective electromechanical constant ($K_{t}^{2}$) of AlN/sapphire-based SAW temperature sensors. Although the impact of acoustic propagation directions on the performance of AlN/sapphire-based SAW resonators at room temperature has been reported [14], the temperature sensor performances, including TCF and temperature dependent *Q* and $K_{t}^{2}$ of SAW resonators along different acoustic propagation directions, have not been studied. Moreover, elastic stiffness [*c*_ij_] with different acoustic propagation directions has been extracted based on finite element method simulation (FEM) without considering the direction-dependent relations of physical parameters, including [*c*_ij_], piezoelectric constants [*e_ik_*] and dielectric constants [*ε_ik_*] along different directions. This extraction method is not physically reasonable. In this paper, we introduce Euler transformation to describe the intrinsic relations of physical parameters along different directions during the extraction of physical parameters. Furthermore, [*c*_ij_], [*e_ik_*] and [*ε_ik_*] along different directions were also extracted in this study. Finally, the possible physical reasons for the origin of anisotropy of [*c*_ij_] along *a*- and *m*-direction of *c*-plane AlN films was analyzed. These experimental and theoretical investigations may be helpful for the development of AlN-based SAW temperature sensors in the future.

## 2. Experimental Methods

**Figure 1:**
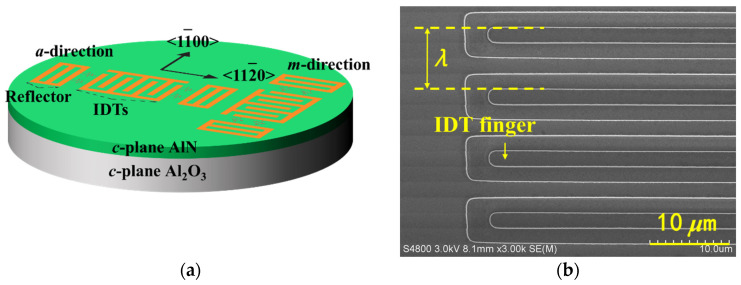
(**a**) Schematic picture of SAW sensors with different directions; (**b**) SEM image of IDT fingers.

We fabricated *c*-plane AlN films with thicknesses of 0.8 and 1.2 μm on 2-inch (0001) sapphire wafers. Recipes for radio frequency (RF) magnetron sputtering-grown AlN films on sapphire have been reported previously [14]. Figure 1a shows the schematic picture of fabricated SAW sensors composed of one interdigital transducer (IDT) and a pair of reflectors. SAW sensors were fabricated via electron beam evaporation and a lift-off photolithography process on prepared AlN films. Ti/Al (10 nm/490 nm) film was deposited on top of the AlN film to form IDT electrodes. The wavelength (*λ*) of the SAW sensors was 8 μm, and the metallization ratio of IDT electrodes was 50%, as shown in Figure 1b. The acoustic wave aperture was 30 *λ*, and the number of short-connected gratings as reflectors on each side of IDTs was 100. To study the impact of the thickness of AlN film (*h*_AlN_), number of IDTs (*N*_IDT_), and acoustic propagation direction on the performance of SAW sensors, various device structures shown in Table 1 were fabricated.

The crystallographic structure and surface morphology of AlN films were characterized by high resolution X-ray diffraction (HRXRD, Bede D1) and atomic force microscopy (AFM, Veeco D3100), respectively. The devices on the wafer were tested in a chamber of a variable temperature probe station (MPI TS2000-SE) at temperatures from −50 °C to 250 °C. The atmosphere in the chamber during the test was nitrogen. The S parameters of devices were measured using a vector network analyzer (VNA, Agilent E8358A) after standard TSOM (through, short, open and match) calibration.

## 3. Results and Discussion

**Figure 2:**
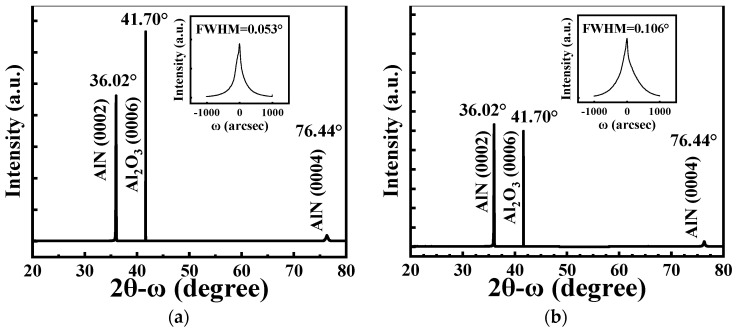
The 2*θ−ω* XRD scan patterns of AlN films with thicknesses of (**a**) 0.8 μm and (**b**) 1.2 μm. The inset shows XRD rocking curves of AlN (0002) films.

Figure 2a,b show the 2*θ − ω* XRD scan patterns of AlN films with thicknesses of 0.8 and 1.2 μm on sapphire, respectively. For all samples, three pronounced diffraction peaks at 2*θ* = 36.02°, 41.70° and 76.44° appeared, corresponding to the hexagonal AlN (0002) plane, Al_2_O_3_ (0006) and AlN (0004) planes, respectively. The AlN (0002) peak together with the detection of high-order AlN (0004) reflection attests to the high crystalline quality of the AlN films sputtered on Al_2_O_3_ substrates [15]. With increasing thickness, the intensity of the AlN (0002) peak significantly increased relative to that of the (0006) plane of Al_2_O_3_. The results indicate that highly c-axis-textured AlN thin films have been successfully grown on sapphire substrates. Inset figures of Figure 2a,b present the XRD rocking curve of the AlN (0002) peak. The full widths at half maximum (FWHM) values of XRD rocking curve of the AlN (0002) peak for films with thicknesses of 0.8 and 1.2 μm were 0.053° and 0.106°, respectively.

**Figure 3:**
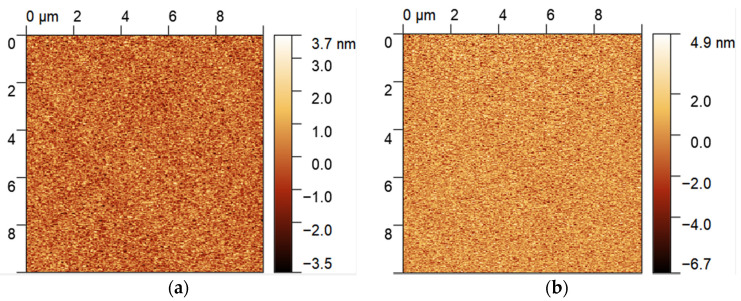
AFM images of AlN films with thicknesses of (**a**) 0.8 μm and (**b**) 1.2 μm in a range of 10 × 10 μm.

Figure 3a,b show the AFM images of AlN films with thicknesses of 0.8 and 1.2 μm, respectively. The two AlN films exhibited smooth surface morphology, with a surface roughness of 0.82 and 1.24 nm, respectively.

**Figure 4:**
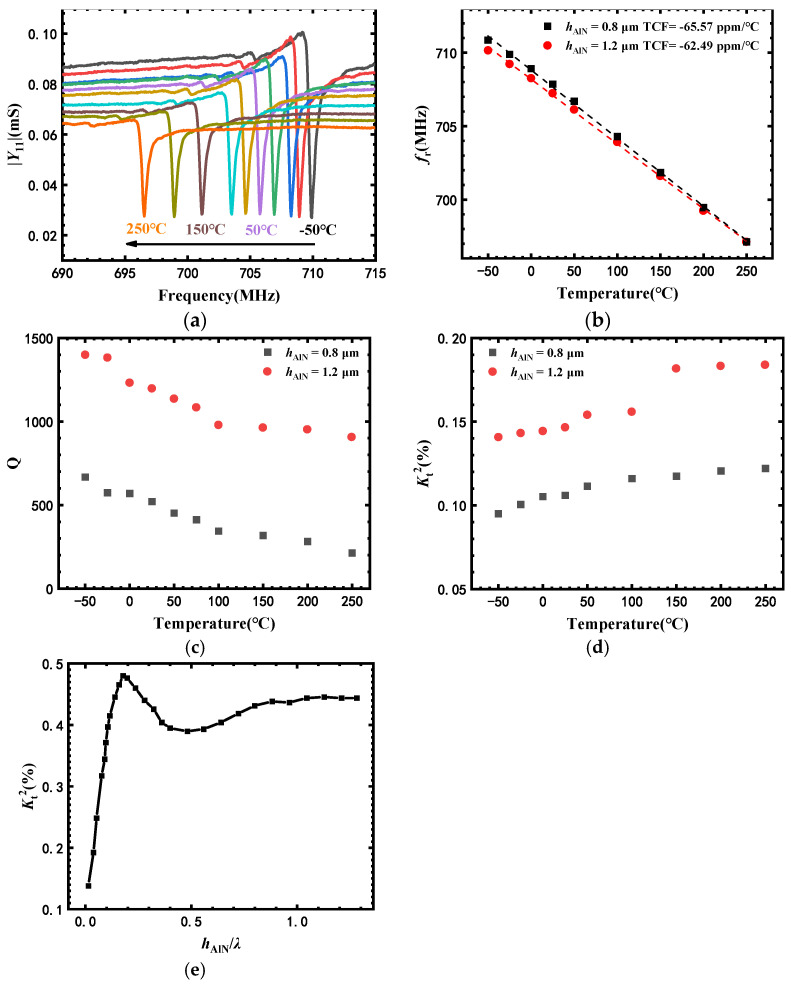
(**a**) Admittance magnitude |*Y*_11_| of SAW-B (*h*_AlN_ = 1.2 μm) versus frequency with various temperatures from −50 °C to 250 °C. Temperature dependency of (**b**) f_r_, (**c**) Q, and (**d**) K_t_^2^ of sensors with different AlN film thicknesses; (**e**) dependence of K_t_^2^ of sensors on *h*_AlN_/λ (λ = 8 μm) by FEM simulation.

Figure 4a shows the admittance magnitude |*Y*_11_| of SAW-B (*h*_AlN_ = 1.2 μm) with device parameters listed in Table 1, versus frequency with various temperatures from −50 °C to 250 °C. The resonant frequency (f_r_) of SAW-B shifted from 709.95 MHz at −50 °C to 696.55 MHz at 250 °C, a 1.89% decrease in frequency. Figure 4b shows the temperature dependency of f_r_ of SAW-A (*h*_AlN_ = 0.8 μm) and SAW-B (*h*_AlN_ = 1.2 μm). The f_r_ of all sensors decreased linearly with increasing temperature, indicating a negative temperature coefficient of elastic stiffness for the AlN/sapphire structure [16,17,18]. The temperature coefficient of frequency (TCF) of sensors is defined by equation

(1) $$TCF=\frac{1}{T-T_{0}}\cdot \frac{f\left(T\right)-f\left(T_{0}\right)}{f\left(T_{0}\right)}$$

where T is the temperature in Celsius, f(T) is the resonance frequency at T, and $T_{0}$ is 25 °C.

The TCFs of SAW-A (*h*_AlN_ = 0.8 μm) and SAW-B (*h*_AlN_ = 1.2 μm) were −65.57 and −62.49 ppm/°C, respectively. As a comparison, the TCF of SAW sensors on bulk AlN is −19 ppm/°C [19]. The negative TCF of SAW sensors based on AlN/sapphire can be attributed to two factors, including the decrease in the phase velocity in the composite structure due to negative temperature coefficient of elastic constants (TCE) and temperature coefficients of expansion of AlN/sapphire, and the increase in *λ* due to thermal expansion [19].

The results indicate that a reduction in *h*_AlN_/*λ* favors the improvement of the TCF of sensors based on AlN/sapphire. For SAW-A and SAW-B, *h*_AlN_/*λ* were 0.1 and 0.15, respectively. The TCF of SAW-A was larger than that of SAW-B, because more surface acoustic waves propagated through the sapphire substrates in SAW-A than in SAW-B. The TCFs of SAW-A and SAW-B were more than two times greater than the estimated TCF of bulk AlN-based SAW, indicating that both AlN and sapphire exhibited a negative temperature coefficient of elastic constants (TCE), and the TCE of sapphire was larger than that of AlN [18]. The TCE of sapphire for *c*_11_, *c*_12_, *c*_13_, *c*_14_, *c*_33_ and *c*_44_ were −75, 40, −80, −70, −85 and −180 ppm/°C, respectively. The TCE of AlN for *c*_11_, *c*_12_, *c*_13_, *c*_33_ and *c*_44_ were −28, −35, −40, −30 and −11 ppm/°C, respectively [18].

Figure 4c,d show the temperature dependency of the quality factor (*Q*) and the electromechanical coupling coefficient $\left(K_{t}^{2}\right)$ of sensors with different AlN film thicknesses. *Q* and $K_{t}^{2}$ were calculated based on the equations reported in [14]. The *Q* of sensors is defined by the Equation (2)

(2) $$Q=\frac{f_{r}}{2}\cdot {\left|\frac{d\phi }{df}\right|}_{f_{r}}$$

where *f*_r_ is the resonant frequency and ${\left|\frac{d\phi }{df}\right|}_{f_{r}}$ is the slope of the phase of the input admittance with respect to the resonant frequency. The *Q* values of SAW-A (*h*_AlN_ = 0.8 μm) and SAW-B (*h*_AlN_ = 1.2 μm) were 520 and 1198, respectively. The $K_{t}^{2}$ values of SAW-A and SAW-B were 0.106% and 0.147%, respectively, indicating that $K_{t}^{2}$ of sensors increased with increase in *h*_AlN_. With increasing temperature, the *Q* decreased and the $K_{t}^{2}$ increased for the sensors. For example, when the temperature increased from −50 °C to 250 °C, the *Q* of SAW-B decreased from 1399 to 906, and its $K_{t}^{2}$ value increased from 0.141% to 0.184%. The decrease in *Q* can be attributed to the increase in acoustic propagation loss with increasing temperature. On the other hand, the relation between $K_{t}^{2}$ and material parameters can be expressed by Equation (3)

(3) $$K_{t}^{2}=\frac{e^{2}\cdot c}{\varepsilon_{0}\cdot \varepsilon }$$

where *e*, *c* and *ε* are the piezoelectric constant, elastic stiffness, and relative dielectric constant of AlN [20], respectively. Because as temperature increases, *ε* increases [21], and *c* decreases [16,21], the increase in $K_{t}^{2}$ can be attributed to the increase in *e* of AlN [22].

**Figure 5:**
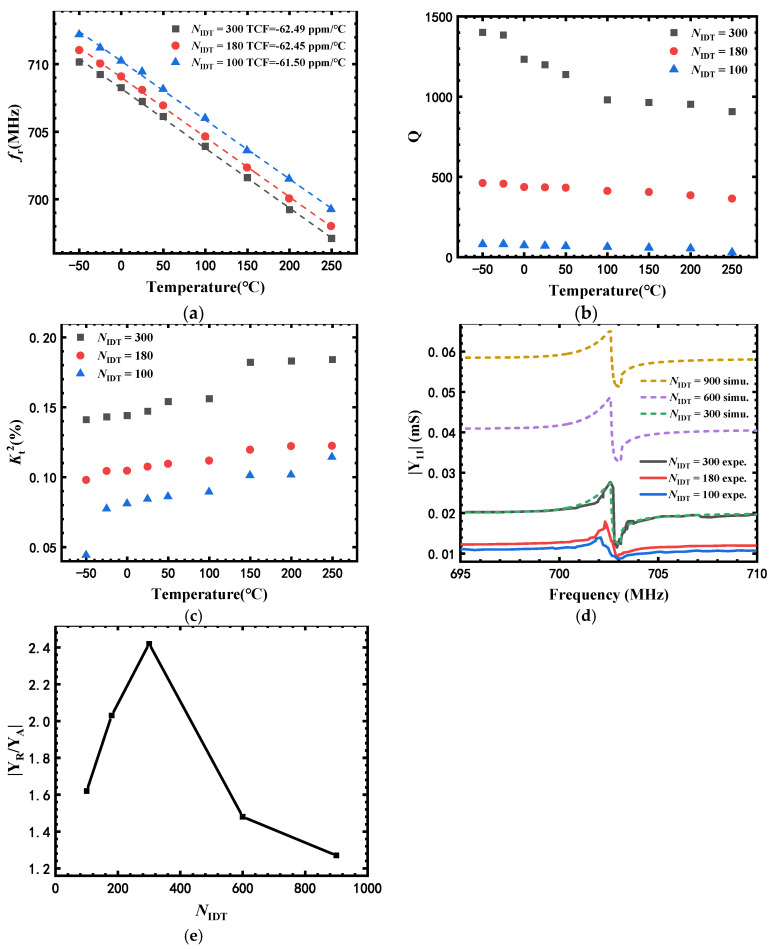
Temperature dependency of (**a**) *f*_r_, (**b**) Q, and (**c**) $K_{t}^{2}$ of sensors with different *N*_IDT_; (**d**) admittance of SAW resonators with *N*_IDT_ of 100, 180 and 300 (experimental results), and with *N*_IDT_ of 300, 600 and 900 (simulated results); (**e**) ratio of conductance at resonant frequency and anti-resonant frequency (*Y*_r_/*Y*_a_) with different *N*_IDT_.

The impact of *h*_AlN_ on $K_{t}^{2}$ of SAW sensors was investigated based on finite element method (FEM) simulation. The thickness of Al electrodes was 300 nm, and the *h*_AlN_ varied from 0.08 to 10.4 μm, corresponding to *h*_AlN_/*λ* values from 0.01 to 1.3. Periodic boundary conditions were applied to the left and right boundaries of the AlN/sapphire structure, while the bottom of the sapphire substrate was fixed. The density of AlN was 3300 kg·m^−3^. Sapphire was treated as an isotropic material with a Young’s modulus of 360 GPa, Poisson’s ratio of 0.22, and density of 3965 kg·m^−3^. Figure 5e shows the dependence of $K_{t}^{2}$ on *h*_AlN_/*λ* (*λ* = 8 μm) by FEM simulation. The value of $K_{t}^{2}$ increased rapidly with the increase in *h*_AlN_/*λ* from 0.01 to 0.18, which is consistent with our experimental results. However, $K_{t}^{2}$ decreased a little with the increase in *h*_AlN_/*λ* from 0.18 to 0.5. When *h*_AlN_/*λ* further increased, $K_{t}^{2}$ increased and gradually saturated.

Figure 5a–c show the temperature dependency of *f*_r_, *Q* and $K_{t}^{2}$ of sensors with different *N*_IDT_, respectively. The TCFs of SAW-B (*N*_IDT_ = 300), SAW-C (*N*_IDT_ = 180) and SAW-D (*N*_IDT_ = 100) were −61.50, −62.45 and −62.49 ppm/°C, respectively, indicating that *N*_IDT_ had a negligible impact on TCF. However, SAW-B exhibited higher *Q* and $K_{t}^{2}$ than those of SAW-C and SAW-D at all temperatures measured. For example, the *Q* and $K_{t}^{2}$ of SAW-B were 1198 and 0.147%, which were 1636% and 74% higher than those of SAW-D at 25 °C. *N*_IDT_ larger than 300 was necessary for AlN/sapphire-based SAW sensors with an acoustic wave aperture of 30 *λ* and a *λ* of 8 μm to guarantee good *Q* values.

We investigated whether the performance of the SAW resonators could be further optimized as *N*_IDT_ continues to increase. The performance of SAW resonators with *N*_IDT_ = 600 and 900 were simulated based on the coupling of modes (COM) model. The COM parameters were extracted from the experimental results of SAW resonators with *N*_IDT_ = 300. Figure 5d shows admittance of SAW resonators with *N*_IDT_ of 100, 180 and 300 (experimental results), and with *N*_IDT_ of 600 and 900 (simulated results). Figure 5e shows the ratio of conductance at resonant frequency and anti-resonant frequency (*Y*_r_/*Y*_a_) with different *N*_IDT_. The value of *Y*_r_/*Y*_a_ increased as *N*_IDT_ increased from 100 to 300. However, with further increase in *N*_IDT_ to 600 and 900, the value of *Y*_r_/*Y*_a_ decreased. In the case of the SAW resonators with very large *N*_IDT_, the devices worked more like large capacitors, resulting in a decrease in *Y*_r_/*Y*_a_.

**Figure 6:**
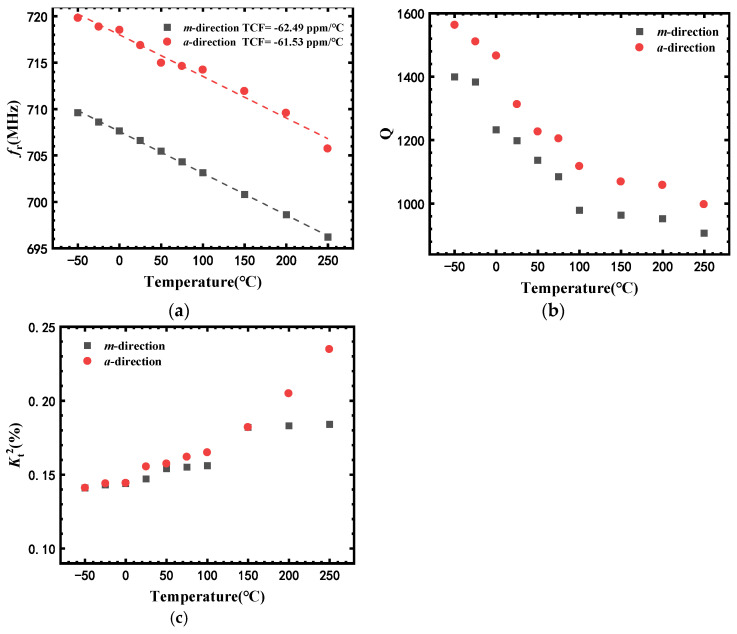
Temperature dependency of (**a**) *f*_r_, (**b**) *Q*, and (**c**) $K_{t}^{2}$ of sensors with different acoustic propagation directions.

Figure 6a–c show the temperature dependency of *f*_r_, *Q*, and $K_{t}^{2}$ of sensors with different acoustic propagation directions. SAW-E (*a*-direction) exhibited higher *f*_r_, *Q* and $K_{t}^{2}$ than those of SAW-B (*m*-direction) at all the temperatures measured. For example, at 25 °C, the *f*_r_, *Q* and $K_{t}^{2}$ of SAW-E (*a*-direction) were 716.9 MHz, 1313 and 0.155%, which were 1.4%, 9.5%, and 5.4% higher than those of SAW-B (*m*-direction). Moreover, the TCF values of SAW-B (*m*-direction) and SAW-E (*a*-direction) were similar, which were −62.49 and −61.53 ppm/°C, respectively.

The higher *f*_r_ of SAW sensors along the *a*-direction relative to those along the *m*-direction can be attributed to their different elastic stiffness. The elastic stiffness of AlN along *a*- and *m*-directions has been reported previously [14]. However, the direction dependency of material parameter-tensors, including elastic stiffness [*c_ij_*], piezoelectric constants [*e_ik_*] and dielectric constants [*ε_ik_*], is totally ignored, which is not physically reasonable [21]. In this paper, [*c_ij_*] was extracted by fitting coupling of modes (COM) model simulation to experimental results of sensors along different directions, considering Euler transformation of parameter-tensors [23].

**Figure 7:**
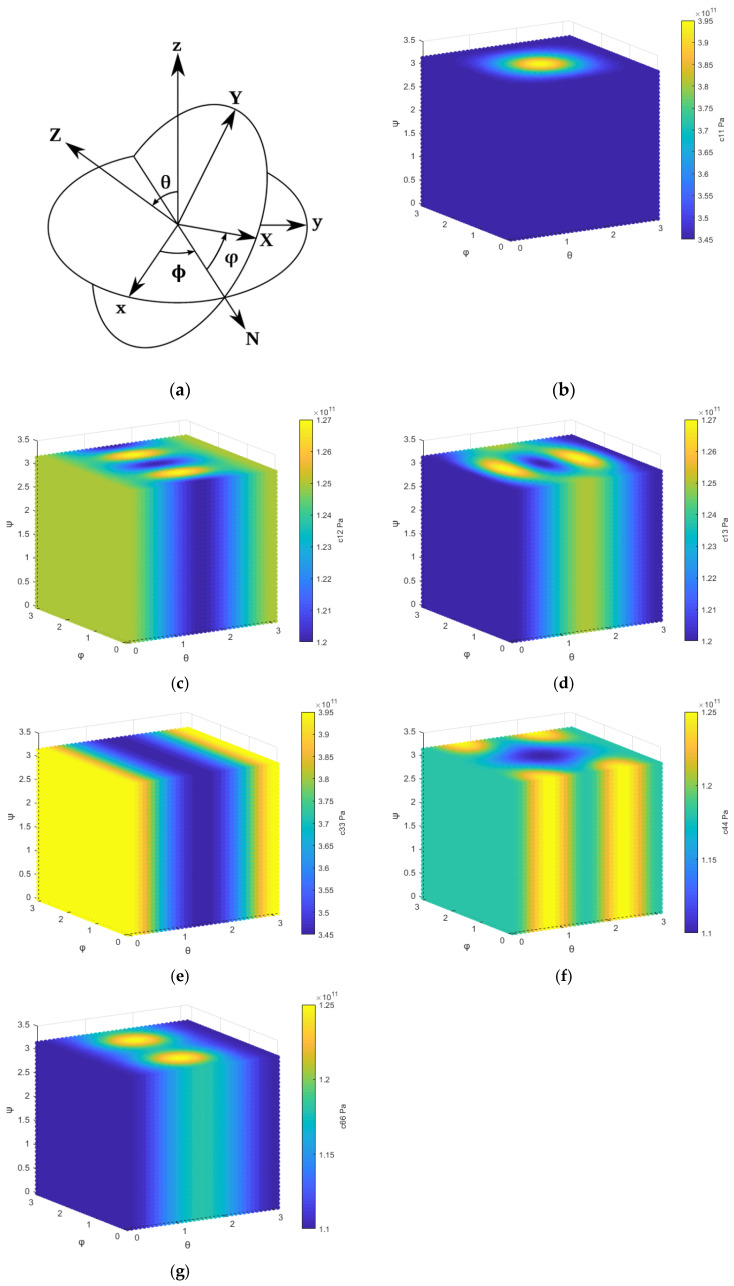
(**a**) Euler angle for right-hand axis system. Numerical distribution of the independent components of the elastic stiffness ([*c_ij_*]) in the Euler angle space, which are (**b**) *c*_11_, (**c**) *c*_12_, (**d**) *c*_13_, (**e**) *c*_33_, (**f**) *c*_44_ and (**g**) *c*_66_, respectively.

The Euler angle is a well-known axis transformation method [23]. Figure 7a shows the Euler angle for the right-hand axis system. Original crystal axes are given by (x, y, z), and the modified new axes are given by (X, Y, Z). The transformation methods for material parameters from the original axes (x, y, z) to the new axes (X, Y, Z) using Euler angle (ϕ, θ, φ) are shown in Equations (4)–(6),

(4) $${\left[c_{ij}\right]}^{\prime }={\left[\alpha \right]}^{-1}\left[c_{ij}\right]\left[\beta \right]$$

(5) $${\left[e_{ik}\right]}^{\prime }={\left[\gamma \right]}^{-1}\left[e_{ik}\right]\left[\beta \right]$$

(6) $${\left[\varepsilon_{ik}\right]}^{\prime }={\left[\gamma \right]}^{-1}\left[\varepsilon_{ik}\right]\left[\gamma \right]$$

where

(7) $$\alpha =\left[\begin{array}{cccccc} l_{1}^{2} & l_{2}^{2} & l_{3}^{2} & 2l_{2}l_{3} & 2l_{3}l_{1} & 2l_{1}l_{2}\\ m_{1}^{2} & m_{2}^{2} & m_{3}^{2} & 2m_{2}m_{3} & 2m_{3}m_{1} & 2m_{1}m_{2}\\ n_{1}^{2} & n_{2}^{2} & n_{3}^{2} & 2n_{2}n_{3} & 2n_{3}n_{1} & 2n_{1}n_{2}\\ m_{1}n_{1} & m_{2}n_{2} & m_{3}n_{3} & m_{2}n_{3}+m_{3}n_{2} & m_{3}n_{1}+m_{1}n_{3} & m_{1}n_{2}+m_{2}n_{1}\\ n_{1}l_{1} & n_{2}l_{2} & n_{3}l_{3} & n_{2}l_{3}+n_{3}l_{2} & n_{3}l_{1}+n_{1}l_{3} & n_{1}l_{2}+n_{2}l_{1}\\ l_{1}m_{1} & l_{2}m_{2} & l_{3}m_{3} & l_{2}m_{3}+l_{3}m_{2} & l_{3}m_{1}+l_{1}m_{3} & l_{1}m_{2}+l_{2}m_{1} \end{array}\right]\;$$

(8) $$\beta =\left[\begin{array}{cccccc} l_{1}^{2} & l_{2}^{2} & l_{3}^{2} & l_{2}l_{3} & l_{3}l_{1} & l_{1}l_{2}\\ m_{1}^{2} & m_{2}^{2} & m_{3}^{2} & m_{2}m_{3} & m_{3}m_{1} & m_{1}m_{2}\\ n_{1}^{2} & n_{2}^{2} & n_{3}^{2} & n_{2}n_{3} & n_{3}n_{1} & n_{1}n_{2}\\ 2m_{1}n_{1} & 2m_{2}n_{2} & 2m_{3}n_{3} & m_{2}n_{3}+m_{3}n_{2} & m_{3}n_{1}+m_{1}n_{3} & m_{1}n_{2}+m_{2}n_{1}\\ 2n_{1}l_{1} & 2n_{2}l_{2} & 2n_{3}l_{3} & n_{2}l_{3}+n_{3}l_{2} & n_{3}l_{1}+n_{1}l_{3} & n_{1}l_{2}+n_{2}l_{1}\\ 2l_{1}m_{1} & 2l_{2}m_{2} & 2l_{3}m_{3} & l_{2}m_{3}+l_{3}m_{2} & l_{3}m_{1}+l_{1}m_{3} & l_{1}m_{2}+l_{2}m_{1} \end{array}\right]$$

(9) $$\gamma =\left[\begin{array}{ccc} l_{1} & l_{2} & l_{3}\\ m_{1} & m_{2} & m_{3}\\ n_{1} & n_{2} & n_{3} \end{array}\right]\;$$

(10) $$\begin{array}{c} l_{1}=\text{cos}\left(\varphi \right)\text{cos}\left(\phi \right)-\text{cos}\left(\theta \right)\text{sin}\left(\phi \right)\text{sin}\left(\varphi \right)\\ l_{2}=-\text{sin}\left(\varphi \right)\text{cos}\left(\phi \right)-\text{cos}\left(\theta \right)\text{sin}\left(\phi \right)\text{cos}\left(\varphi \right)\\ l_{3}=\text{sin}\left(\theta \right)\text{sin}\left(\phi \right)\\ m_{1}=\text{cos}\left(\varphi \right)\text{sin}\left(\phi \right)+\text{cos}\left(\theta \right)\text{cos}\left(\phi \right)\text{sin}\left(\varphi \right)\\ m_{2}=-\text{sin}\left(\varphi \right)\text{sin}\left(\phi \right)+\text{cos}\left(\theta \right)\text{cos}\left(\phi \right)\text{cos}\left(\varphi \right)\\ m_{3}=-\text{sin}\left(\theta \right)\text{cos}\left(\phi \right)\\ n_{1}=\text{sin}\left(\varphi \right)\text{sin}\left(\theta \right)\\ n_{2}=\text{cos}\left(\varphi \right)\text{sin}\left(\theta \right)\\ n_{3}=\text{cos}\left(\theta \right) \end{array}$$

Figure 7b–g show the variation in each independent component of the elastic stiffness tensor in the Euler angle space using previously reported [*c_ij_*] [17,24]. Considering the symmetry of the Euler transformation, it is sufficient to study the variation in *ϕ, θ,* and *φ* between 0 and π. We can observe that once the [*c_ij_*] along the *m*-direction is defined, the [*c_ij_*] along other directions is determined by Euler transformation. This is also valid for other tensor parameters such as [*e_ik_*] and [*ε_ik_*]. For an ideal *c*-plane, the [*c_ij_*] will not change in any value of φ, as shown in Figure 7b–g. However, an offset angle of sputtered AlN film exists, considering the offset angle of *c*-plane sapphire. We introduced two variables, including Δϕ and Δθ, to describe the offset angle from ideal *c*-plane. Moreover, the frequency difference in orientations may be attributed to mismatch between the lattice constant, poly-crystalline structure of AlN film, amorphous structure in the interface of substrate and AlN film.

**Figure 8:**
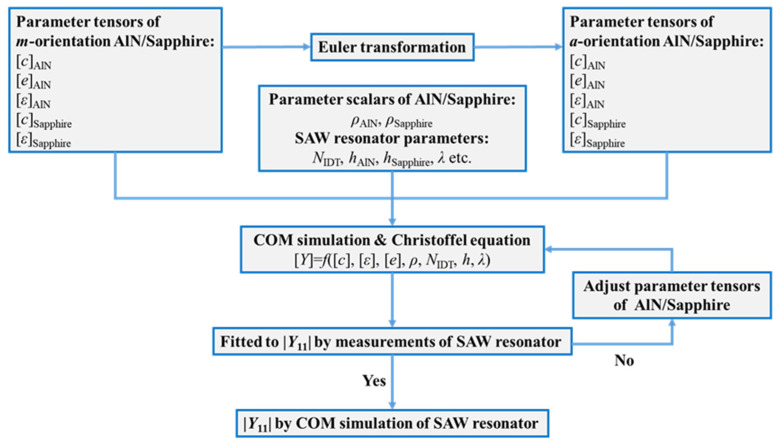
Flowchart of the simulation technique implemented, combining Euler transformation with COM theory.

Figure 8 shows the flowchart of the simulation technique implemented, combining Euler transformation and COM theory with the Christoffel equation to describe the boundary conditions of AlN/sapphire based-SAW sensors [25,26]. Device and material parameters for the simulation can be divided into tensors and scalars. [*c_ij_*]*,* [*e_ik_*] and [*ε_ik_*] are tensors that change with direction and can be described by the Euler transformation discussed above. Density (*ρ*), *h*, *N*_IDT_, *λ*, etc., are scalars, which are constants.

**Figure 9:**
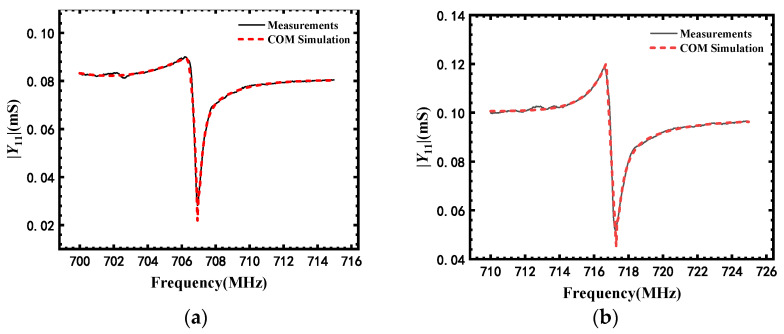
Admittance extracted from the COM simulation compared with the experimental results of sensors along (**a**) *m*-and (**b**) *a*-directions at 25 °C.

First, we performed a COM simulation for the SAW sensor along the *m*-direction; the reported material parameters reported in [17,24] were used as the input parameters. Except for [*c_ij_*] of AlN film which was extracted, other input parameters are listed in Table 2 [25]. Then the |*Y*_11_| from the COM simulation was adjusted by modifying the tensors of AlN until it fitted well with the measured |*Y*_11_| of sensors along the *m*-direction, as shown in Figure 9a. Subsequently, [*e_ik_*], [*ε_ik_*] and extracted [*c_ij_*] along the *m*-direction were changed to those along the *a*-direction by Euler transformation and were used for the COM simulation of sensors along the *a*-direction. If the simulation results did not fit well with the measured |*Y*_11_| along the *a*-direction, [*c_ij_*] was adjusted until both the COM simulated |*Y*_11_| of *m*-direction and *a*-direction fitted well with their measured |*Y*_11_|, as shown in Figure 9a,b. In our simulation, when Δϕ and Δθ are 2° and 4°, respectively, the COM simulation and our measurement results fit well, as shown in Figure 9a,b. The [*c*_ij_] values of AlN along the *m*- and *a*-directions are listed in Table 2. The higher *f*_r_ of sensors along *a*-direction relative to those along the *m*-direction can be attributed to higher *c*_11_, *c*_22_, *c*_44_, and *c*_55_ values.

## 4. Conclusions

In conclusion, we studied the impact of *h*_AlN_, *N*_IDT_, and acoustic propagation direction on TCF, *Q*, and $K_{t}^{2}$ of SAW temperature sensors on *c*-plane AlN/sapphire. The FWHM values of the XRD rocking curve of the AlN (0002) peak for films with thicknesses of 0.8 and 1.2 μm were 0.053° and 0.106°, respectively. The surface roughness of films with thicknesses of 0.8 and 1.2 μm was 0.82 and 1.24 nm, respectively. For all sensors measured from −50 °C to 250 °C, *f*_r_ decreased linearly, *Q* decreased due to the increase in acoustic propagation loss, and $K_{t}^{2}$ increased due to the increase in piezoelectric constants with increasing temperature. The TCFs of sensors on AlN films with thicknesses of 0.8 and 1.2 μm were −65.57 and −62.49 ppm/°C, respectively. The acoustic propagation direction and *N*_IDT_ did not obviously impact the TCF of sensors, but did impact the *Q* and $K_{t}^{2}$ of sensors significantly. The *Q* and $K_{t}^{2}$ of sensors with *N*_IDT_ of 300 were 1198 and 0.147 %, which were 1636% and 74% higher than those with *N*_IDT_ of 100 at 25 °C. The *f*_r_, *Q* and $K_{t}^{2}$ of sensors along the *a*-direction were 716.9 MHz, 1313 and 0.155%, which were 1.4%, 9.5%, and 5.4% higher than that along the *m*-direction at 25 °C. Finally, the [*c_ij_*], [*e_ik_*] and [*ε_ik_*] of AlN were extracted by fitting the COM model simulation to experimental results of sensors along different directions considering Euler transformation. The higher *f*_r_ of the sensors along the *a*-direction relative to those along the *m*-direction can be attributed to its larger *c*_11_, *c*_22_, *c*_44_, and *c*_55_ values.

## Figures and Tables

**Table 1:** Device parameters of fabricated AlN/sapphire-based SAW sensors.

Sample	Thickness of AlN *(h*_AlN_*)/*μm	Number of IDT *(N*_IDT_*)*	Direction
A	0.8	300	*m*
B	1.2	300	*m*
C	1.2	180	*m*
D	1.2	100	*m*
E	1.2	300	*a*

**Table 2:** Physical constants of AlN and sapphire of *m*- and *a*-directions (T = 25 °C).

Direction	*m*	*a*
[*c*]_AlN_ (Gpa)	$\left[\begin{array}{cccccc} 345 & 125 & 120 & 0 & 0 & 0\\ 125 & 345 & 120 & 0 & 0 & 0\\ 120 & 120 & 395 & 0 & 0 & 0\\ 0 & 0 & 0 & 118 & 0 & 0\\ 0 & 0 & 0 & 0 & 118 & 0\\ 0 & 0 & 0 & 0 & 0 & 110 \end{array}\right]$	$\left[\begin{array}{cccccc} 405 & 125 & 120 & 0 & 0 & 0\\ 125 & 405 & 120 & 0 & 0 & 0\\ 120 & 120 & 395 & 0 & 0 & 0\\ 0 & 0 & 0 & 140 & 0 & 0\\ 0 & 0 & 0 & 0 & 140 & 0\\ 0 & 0 & 0 & 0 & 0 & 110 \end{array}\right]$
[*e*]_AlN_ (C/m^2^)	$\left[\begin{array}{cccccc} 0 & 0 & 0 & 0 & -0.48 & 0\\ 0 & 0 & 0 & -0.48 & 0 & 0\\ -0.58 & -0.58 & 1.55 & 0 & 0 & 0 \end{array}\right]$	$\left[\begin{array}{cccccc} 0 & 0 & 0 & 0 & -0.5 & 0\\ 0 & 0 & 0 & -0.5 & 0 & 0\\ -0.55 & -0.55 & 1.55 & 0 & 0 & 0 \end{array}\right]$
[*ε*]_AlN_ (10^−11^F/m)	$\left[\begin{array}{ccc} 8 & 0 & 0\\ 0 & 8 & 0\\ 0 & 0 & 9.5 \end{array}\right]$	$\left[\begin{array}{ccc} 8 & 0 & 0\\ 0 & 8 & 0\\ 0 & 0 & 9.5 \end{array}\right]$
[*c*]_sapphire_ (Gpa)	$\left[\begin{array}{cccccc} 497 & 164 & 111 & -23.5 & 0 & 0\\ 164 & 497 & 111 & 0 & 0 & 0\\ 111 & 111 & 498 & 0 & 0 & 0\\ -23.5 & 0 & 0 & 147 & 0 & 0\\ 0 & 0 & 0 & 0 & 147 & 0\\ 0 & 0 & 0 & 0 & 0 & 0 \end{array}\right]$	$\left[\begin{array}{cccccc} 497 & 164 & 111 & -23.5 & 0 & 0\\ 164 & 497 & 111 & 0 & 0 & 0\\ 111 & 111 & 498 & 0 & 0 & 0\\ -23.5 & 0 & 0 & 147 & 0 & 0\\ 0 & 0 & 0 & 0 & 147 & 0\\ 0 & 0 & 0 & 0 & 0 & 0 \end{array}\right]$
[*ε*]_sapphire_ (10^−11^F/m)	$\left[\begin{array}{ccc} 8.28 & 0 & 0\\ 0 & 8.28 & 0\\ 0 & 0 & 0.12 \end{array}\right]$	$\left[\begin{array}{ccc} 8.28 & 0 & 0\\ 0 & 8.28 & 0\\ 0 & 0 & 0.12 \end{array}\right]$

## Data Availability

The data presented in this paper is available from the corresponding author upon request.
